# A novel predictive model of hospital stay for Total Knee Arthroplasty patients

**DOI:** 10.3389/fsurg.2022.807467

**Published:** 2023-01-06

**Authors:** Bo Liu, Yijiang Ma, Chunxiao Zhou, Zhijie Wang, Qiang Zhang

**Affiliations:** ^1^ Department of Orthopaedics, Beijing Ditan Hospital, Capital Medical University, Beijing, China; ^2^ Department of Respiratory and Critical Care Medicine, The Affiliated Hospital of Qingdao University, Qingdao, China; ^3^ Department of Hematology, The Affiliated Hospital of Qingdao University, Qingdao, China; ^4^ Department of Spinal Surgery, The Affiliated Hospital of Qingdao University, Qingdao, China

**Keywords:** total knee arthroplasty, hospital stay, the risk factor, nomogram, predictive model, a cohort study

## Abstract

**Objective:**

This study aimed to explore the main risk factors affecting Total Knee Arthroplasty (TKA) patients and develop a predictive nomogram of hospital stay.

**Methods:**

In total, 2,622 patients undergoing TKA in Singapore were included in this retrospective cohort study. Hospital extension was defined based on the 75% quartile (Q3) of hospital stay. We randomly divided all patients into two groups using a 7:3 ratio of training and validation groups. We performed univariate analyses of the training group, in which variables with *P*-values < 0.05 were included and then subjected to multivariate analysis. The multivariable logistic regression analysis was applied to build a predicting nomogram, using variable *P*-values < 0.01. To evaluate the prediction ability of the model, we calculated the C-index. The ROC, Calibration, and DCA curves were drawn to assess the model. Finally, we verified the accuracy of the model using the validation group and by also using the C-index. The ROC curve, Calibration curve, and DCA curve were then applied to evaluate the model in the validation group.

**Results:**

The final study included 2,266 patients. The 75% quartile (Q3) of hospital stay was six days. In total, 457 (20.17%) patients had hospital extensions. There were 1,588 patients in the training group and 678 patients in the validation group. Age, Hb, D.M., Operation Duration, Procedure Description, Day of Operation, Repeat Operation, and Blood Transfusion were used to build the prediction model. The C-index was 0.680 (95% CI: 0.734–0.626) in the training group and 0.710 (95% CI: 0.742–0.678) for the validation set. The calibration curve and DCA indicated that the hospital stay extension model showed good performance in the training and validation groups.

**Conclusion:**

To identify patients' risk factors early, medical teams need to plan a patient’s rehabilitation path as a whole. Its advantages lie in better resource allocation, maximizing medical resources, improving the functional recovery of patients, and reducing the overall cost of hospital stay and surgery, and will help clinicians in the future.

## Introduction

Total Knee Arthroplasty (TKA) is the primary surgical procedure for treating severe knee disorders, relieving knee pain, and reestablishing knee function (1). It can help patients restore knee joint function and improve their quality of life (2). The increased life expectancy and aging of society have led to a dramatic increase in the number of patients with knee disorders. TKA is currently the most important and effective way to solve knee disorders, indicating that the demand for TKA will increase dramatically (3, 4). It is estimated that more than 700,000 TKA surgeries are performed in the United States annually and that this number will increase to nearly 3.5 million by 2030. It is therefore important that TKA is performed economically and effectively (5).

The costs of surgical procedures, inpatient care, and postoperative care have substantially increased due to enhanced intra- and peri-operative management (6). Meanwhile, there has been a noticeable decline in the global economy, and medical insurance expenditure has increased significantly. Some countries have removed TKA from the List of Medicare Outpatient Payment System Rules (7). The main focus of attempts to reduce hospitalization costs is to minimize hospital stays as shortening them will reduce medical expenses and society's medical burden (8).

TKA surgery may be accompanied by severe complications, which affect patients' prognosis and increase the possibility of disability and death (9). However, an extended hospital stay is a risk factor for postoperative complications. It is essential to reduce postoperative complications such as postoperative infection, thromboembolism, postoperative delirium, and cognitive dysfunction (10). The main factors of hospital stay, the incidence of surgical complications, hospitalization expenses, and medical resources are intractable issues that should be solved as soon as possible. This study aimed to explore the main risk factors and establish a predictive nomogram of Hospital Stay in undergoing Total Knee Arthroplasty (TKA).

## Patients and methods

### Patient and dates

Our secondary analysis was based on a single-center retrospective cohort study of patients who underwent TKA at Singapore General Hospital from January 2013 to June 2014 (11). We downloaded the raw data uploaded at the “DATADRYAD” website (www.datadryad.org). Abdullah et al. (11) shared the original data to the dryad database. We utilized these data for secondary analysis on a different hypothesis without violating the authors' rights.

The Beijing Ditan Hospital of Capital Medical University Ethics Committee approved our study. The Ethics Committee confirmed that informed consent was not required because the data are available publicly *via* the “DATADRYAD” Website (www.datadryad.org), and data were analyzed anonymously. The ethics committee waived the requirement for informed consent from all patients.

## Methods

The study included 2,622 patients undergoing TKA in Singapore in a retrospective cohort study. We removed patients whose variables contained missing values, for example, BMI, D.M., D.M. on insulin, etc, which meant that 2,266 patients met the criteria and were included in the study. The prolonged hospital stay was defined as over the 75% quartile (Q3) of hospital stay (12–14). We randomly divided all patients into two groups in a 7:3 ratio: training and validation groups. We performed a univariate analysis in the training group, in which variables with *P*-values < 0.05 were included and then subjected to multivariate analysis. Multivariable logistic regression analysis was then applied to build a predicting nomogram using the variables the *P*-values were < 0.01. Furthermore, ROC, Calibration, and DCA curves were drawn to assess the model in the training and validation group.

## Statistical analysis

R software (version 4.1.2) was used for all statistical analyses. (https://www.R-project.org). Using the R package “tableone”, Chi-squared tests (categorical variables) and one-way analysis of variance, or *K*–*W* test (continuous variables of a normal distribution or skewed distribution) we verified significant differences between different groups. Using the R package “caret”, we randomly divided all patients into two groups, using a 7:3 ratio for the training and validation groups. We first conducted univariate analysis, then multivariable regression under the package “rms”. *P*-value < 0.05 (bilateral) was considered statistically significant in the univariate analysis, and a *P*-value < 0.01 (bilateral) was considered statistically significant in the multivariable logistic regression. Predictors with a *P*-value < 0.01 were applied to develop the model that predicted the prolonged hospital stay.

The C-index (the concordance index) was used to evaluate the model's predictive ability. The C-index refers to the proportion of all patients whose predicted outcome is concordant with the actual result. Similarly, the ROC curve was used for the prediction accuracy of X to Y, enabling us to judge whether a specific factor has a diagnostic value for identifying a particular disease. The ROC curve reflects the relationship between sensitivity and specificity (package = “ROCR”). The DCA curve was performed by quantifying the net benefit at different threshold probabilities (package = “rmda”). Furthermore, the ROC curve, the calibration, and the decision curve analysis were used to evaluate the nomograms in the validation group.

## Results

### The demographic characteristics of patients who underwent TKA

**Figure 1:**
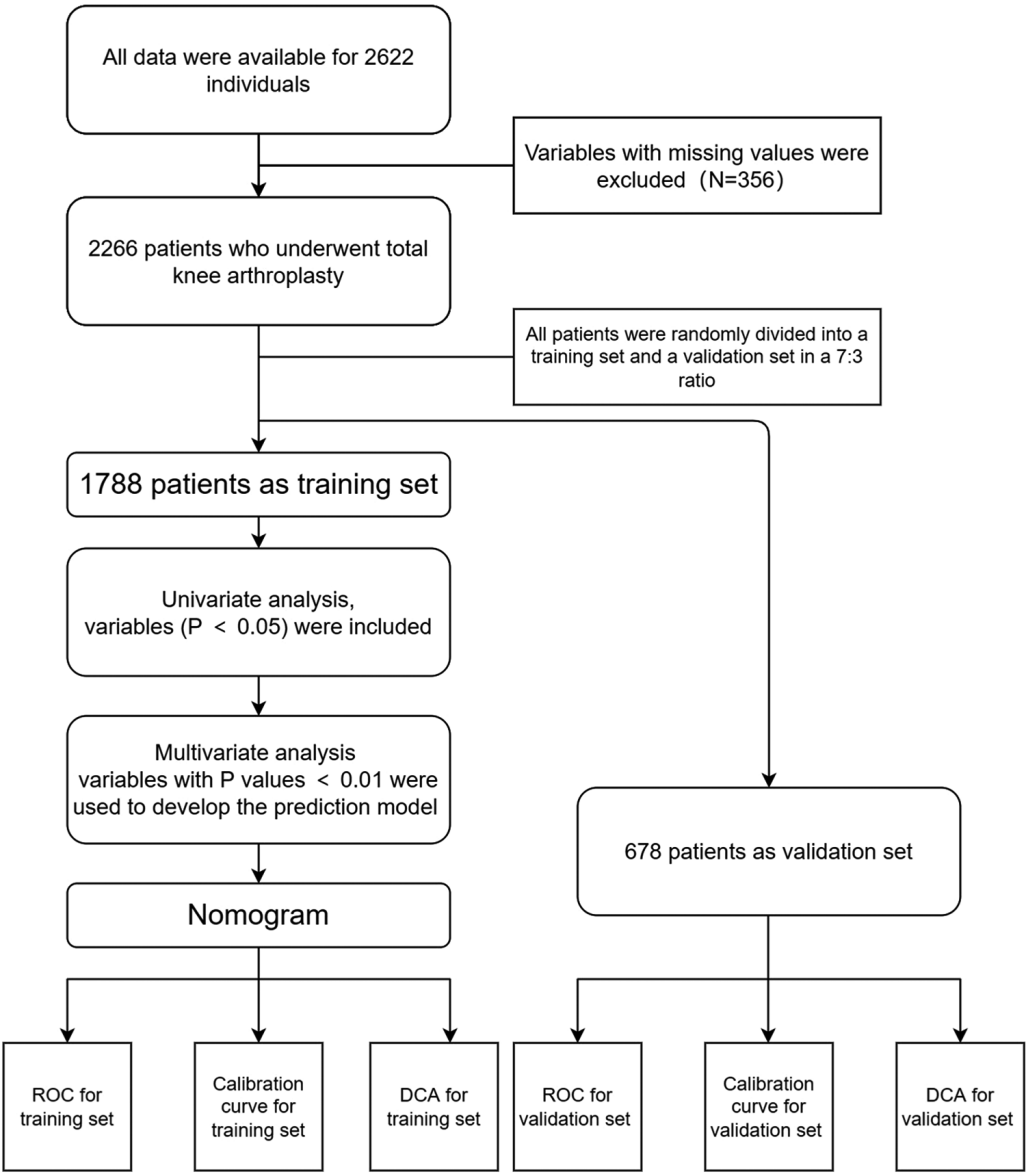
Flowchart of our research.

In total 2,266 of the patients who underwent TKA between January 2013 and June 2014 in the retrospective cohort were included, and 356 patients were excluded because the variable had a missing value (Figure 1). The two groups had no significant differences in BMI, Gender, Smoking habits, and OSA distribution. However, patients had a higher ASA score, longer operation time, higher ratio of bilateral TKA, higher incidence of anemia, higher risk of Blood Transfusion, repeated operation, and general anesthesia in the extended hospital stay group (Table 1). We randomized 1,788 patients into the training group and 678 patients into the validation group. The 75% quartiles (Q3) of hospital stays were six days, with 457 patients (20.17%) hospitalized for over six days (Tables 2, 3).

**Table 1:** The demographic characteristics of patients.

	Overall (*n* = 2266)	Hospital stay ≤6 days (*n* = 1809)	Hospital stay >6 days (*n* = 457)	*P*-value
BMI (mean ± SD)	27.85 ± 5.75	27.89 ± 5.90	27.65 ± 5.14	**0** **.** **422**
Age (mean ± SD)	66.20 ± 8.03	65.76 ± 7.97	67.96 ± 8.03	<0.001
Hb (mean ± SD)	13.10 ± 1.45	13.19 ± 1.39	12.78 ± 1.63	<0.001
Operation_duration_in_mins [median (IQR)]	80.00 [65.00, 100.00]	80.00 [65.00, 95.00]	85.00 [70.00, 105.00]	<0.001
Periop_blood_units (mean ± SD)	0.09 ± 0.47	0.04 ± 0.25	0.28 ± 0.90	<0.001
**ASA_status (%)**				
1	156 (6.9)	126 (7.0)	30 (6.6)	<0.001
2	1,972 (87.0)	1,592 (88.0)	380 (83.2)	
3	138 (6.1)	91 (5.0)	47 (10.3)	
**Race (%)**				
Chinese	1,901 (83.9)	1,514 (83.7)	387 (84.7)	0.014
Indian	134 (5.9)	102 (5.6)	32 (7.0)	
Maly	167 (7.4)	147 (8.1)	20 (4.4)	
Other	64 (2.8)	46 (2.5)	18 (3.9)	
**Gender (%)**				
Male	551 (24.3)	439 (24.3)	112 (24.5)	**0** **.** **963**
Female	1,715 (75.7)	1,370 (75.7)	345 (75.5)	
**Type_of_anaesthesia (%)**				
GA	815 (36.0)	613 (33.9)	202 (44.2)	<0.001
RA	1,426 (62.9)	1,176 (65.0)	250 (54.7)	
GA_RA	25 (1.1)	20 (1.1)	5 (1.1)	
**Procedure_description (%)**				
Unilateral	2,078 (91.7)	1,697 (93.8)	381 (83.4)	<0.001
Bilateral	167 (7.4)	104 (5.7)	63 (13.8)	
Revision	21 (0.9)	8 (0.4)	13 (2.8)	
**Smoking (%)**				
No	2,061 (91.0)	1,648 (91.1)	413 (90.4)	**0** **.** **694**
Yes	205 (9.0)	161 (8.9)	44 (9.6)	
OSA (%)				
No	2,048 (90.4)	1,627 (89.9)	421 (92.1)	**0** **.** **185**
Yes	218 (9.6)	182 (10.1)	36 (7.9)	
**DM (%)**				
No	1,829 (80.7)	1,491 (82.4)	338 (74.0)	<0.001
Yes	437 (19.3)	318 (17.6)	119 (26.0)	
IHD (%)				
No	2,137 (94.3)	1,718 (95.0)	419 (91.7)	**0** **.** **189**
Yes	129 (5.7)	91 (5.0)	38 (8.3)	
**CHF (%)**				
No	2,244 (99.0)	1,796 (99.3)	448 (98.0)	0.03
Yes	22 (1.0)	13 (0.7)	9 (2.0)	
**CVA (%)**				
No	2,221 (98.0)	1,783 (98.6)	438 (95.8)	<0.001
Yes	45 (2.0)	26 (1.4)	19 (4.2)	
**Creatinine_ > _2 mg/dl (%)**				
No	2,247 (99.2)	1,799 (99.4)	448 (98.0)	0.007
Yes	19 (0.8)	10 (0.6)	9 (2.0)	
**Day_operation (%)**				
Mon	368 (16.2)	277 (15.3)	91 (19.9)	0.001
Tue	500 (22.1)	387 (21.4)	113 (24.7)	
Wed	404 (17.8)	321 (17.7)	83 (18.2)	
Thu	516 (22.8)	445 (24.6)	71 (15.5)	
Fri	361 (15.9)	285 (15.8)	76 (16.6)	
Sat	117 (5.2)	94 (5.2)	23 (5.0)	
**Blood_transfusion (%)**				
No	2,143 (94.6)	1,751 (96.8)	392 (85.8)	<0.001
Yes	123 (5.4)	58 (3.2)	65 (14.2)	
**Repeat_Op_within_30_days (%)**				
No	2,250 (99.3)	1,805 (99.8)	445 (97.4)	<0.001
Yes	16 (0.7)	4 (0.2)	12 (2.6)	

**Table 2:** Clinical characteristics of patients who underwent TKA in training and verification group.

	Training Group (*n* = 1588)	Validation Group (*n* = 678)	Overall (*n* = 2266)
BMI (mean ± SD)	27.9 ± 6.15	27.7 ± 4.71	27.8 ± 5.75
Age (mean ± SD)	66.3 ± 8.12	65.9 ± 7.81	66.2 ± 8.03
**ASA_status**			
1	111 (7.0%)	45 (6.6%)	156 (6.9%)
2	1,380 (86.9%)	592 (87.3%)	1,972 (87.0%)
3	97 (6.1%)	41 (6.0%)	138 (6.1%)
Hb (mean ± SD)	13.1 ± 1.48	13.2 ± 1.36	13.1 ± 1.45
Hospital stay in_days (mean ± SD)	5.48 ± 5.15	5.23 ± 3.46	5.41 ± 4.71
**Hospital stay**			
≤6 days	1,258 (79.2%)	551 (81.3%)	1,809 (79.8%)
>6 days	330 (20.8%)	127 (18.7%)	457 (20.2%)
Operation_duration_in_mins (mean ± SD)	84.8 ± 27.9	84.8 ± 27.0	84.8 ± 27.6
**Race**			
Chinese	1,324 (83.4%)	577 (85.1%)	1,901 (83.9%)
Indian	96 (6.0%)	38 (5.6%)	134 (5.9%)
Maly	122 (7.7%)	45 (6.6%)	167 (7.4%)
other	46 (2.9%)	18 (2.7%)	64 (2.8%)
**Gender**			
Male	371 (23.4%)	180 (26.5%)	551 (24.3%)
Female	1,217 (76.6%)	498 (73.5%)	1,715 (75.7%)
**Type_of_anaesthesia**			
GA	576 (36.3%)	239 (35.3%)	815 (36.0%)
RA	997 (62.8%)	429 (63.3%)	1,426 (62.9%)
GA_RA	15 (0.9%)	10 (1.5%)	25 (1.1%)
**Procedure_description**			
Unilateral	1,459 (91.9%)	619 (91.3%)	2,078 (91.7%)
Bilateral	114 (7.2%)	53 (7.8%)	167 (7.4%)
Revision	15 (0.9%)	6 (0.9%)	21 (0.9%)
**Smoking**			
No	1,442 (90.8%)	619 (91.3%)	2,061 (91.0%)
Yes	146 (9.2%)	59 (8.7%)	205 (9.0%)
**OSA**			
No	1,427 (89.9%)	621 (91.6%)	2,048 (90.4%)
Yes	161 (10.1%)	57 (8.4%)	218 (9.6%)
**DM**			
No	1,271 (80.0%)	558 (82.3%)	1,829 (80.7%)
Yes	317 (20.0%)	120 (17.7%)	437 (19.3%)
**IHD**			
No	1,505 (94.8%)	632 (93.2%)	2,137 (94.3%)
Yes	83 (5.2%)	46 (6.8%)	129 (5.7%)
**CHF**			
No	1,573 (99.1%)	671 (99.0%)	2,244 (99.0%)
Yes	15 (0.9%)	7 (1.0%)	22 (1.0%)
**CVA**			
No	1,564 (98.5%)	657 (96.9%)	2,221 (98.0%)
Yes	24 (1.5%)	21 (3.1%)	45 (2.0%)
**Creatinine_ > _2 mg/dl**			
No	1,575 (99.2%)	672 (99.1%)	2,247 (99.2%)
Yes	13 (0.8%)	6 (0.9%)	19 (0.8%)
**Day_operation**			
Mon	256 (16.1%)	112 (16.5%)	368 (16.2%)
Tue	340 (21.4%)	160 (23.6%)	500 (22.1%)
Wed	305 (19.2%)	99 (14.6%)	404 (17.8%)
Thu	349 (22.0%)	167 (24.6%)	516 (22.8%)
Fri	255 (16.1%)	106 (15.6%)	361 (15.9%)
Sat	83 (5.2%)	34 (5.0%)	117 (5.2%)
Periop_blood_units (mean ± SD)	0.100 ± 0.520	0.0634 ± 0.340	0.0891 ± 0.474
**Blood_transfusion**			
No	1,494 (94.1%)	649 (95.7%)	2,143 (94.6%)
Yes	94 (5.9%)	29 (4.3%)	123 (5.4%)
**Repeat_Op_within_30_days**			
No	1,574 (99.1%)	676 (99.7%)	2,250 (99.3%)
Yes	14 (0.9%)	2 (0.3%)	16 (0.7%)

**Table 3:** Univariate and multivariate analysis of prolonged hospital stay in undergoing TKA patients.

	Univariable				Multivariable			
	OR	5%Cl	95%Cl	*P*	OR	5%Cl	95%Cl	*P*
Age (year)	1.04	1.02	1.05	**<0**.**001**	1.04	1.02	1.06	**<0**.**001**
Race								0.089
Chinese	Ref							
Indian	1.11	0.68	1.82	0.683	1.03	0.60	1.77	
Maly	0.52	0.30	0.91	**0**.**022**	0.52	0.28	0.96	
other	1.47	0.76	2.83	0.250	1.51	0.75	3.05	
Hb	0.81	0.74	0.88	**<0**.**001**	0.83	0.76	0.91	**<0**.**001**
BMI (kg/m^2^)	0.98	0.96	1.01	0.224				
**Gender**								
Female	Ref							
Male	1.02	0.77	1.36	0.873				
ASA_status								0.485
1	Ref							
2	1.15	0.70	1.90	0.590	0.98	0.57	1.68	
3	2.24	1.18	4.26	**0**.**014**	1.34	0.66	2.73	
Operation_duration_in_mins	1.01	1.01	1.02	**<0**.**001**	1.0084	1.0031	1.0137	**0**.**002**
Type_of_anaesthesia								0.035
GA	Ref							
RA	0.65	0.51	0.84	**<0**.**001**	0.69	0.53	0.91	
GA_RA	0.74	0.20	2.65	0.639	0.77	0.20	2.89	
Procedure_description							** **	**0**.**003**
Unilateral	Ref							
Bilateral	2.67	1.79	3.98	**<0**.**001**	1.99	1.18	3.37	
Revision	4.86	1.75	13.5	**0**.**002**	4.33	1.40	13.38	
**Smoking**								
No	Ref							
Yes	1.18	0.78	1.76	0.434				
**OSA**								
No	Ref							
Yes	0.82	0.54	1.25	0.362				
**DM**								
No	Ref							
Yes	1.81	1.37	2.40	**<0**.**001**	1.72	1.27	2.33	**<0**.**001**
**IHD**								
No	Ref							
Yes	1.4	0.85	2.32	0.189				
**CHF**								
No	Ref							
Yes	3.39	1.22	9.41	**0**.**019**	1.87	0.60	5.85	0.281
**CVA**								
No	Ref							
Yes	2.78	1.22	6.31	**0**.**015**	2.95	1.17	7.42	0.021
**Creatinine_ > _2 mg/dl**								
No	Ref							
Yes	3.31	1.10	9.92	**0**.**033**	1.94	0.55	6.82	0.301
Day_operation								**0**.**002**
Mon	Ref							
Tue	0.89	0.61	1.30	0.543	1.1	(0.72,1.66)	0.665	
Wed	0.81	0.55	1.20	0.296	0.77	0.50	1.18	0.235
Thu	0.49	0.33	0.74	**<0**.**001**	0.47	0.30	0.74	**<0**.**001**
Fri	0.83	0.55	1.24	0.362	0.80	0.50	1.25	0.32
Sat	0.6	0.31	1.13	0.113	0.74	0.38	1.46	0.388
**Blood_transfusion**								
No	Ref							
Yes	4.93	3.22	7.54	**<0**.**001**	2.80	1.74	4.51	**<0**.**001**
**Repeat_Op_within_30_days**								
No	Ref							
Yes	9.8	3.05	31.44	**<0**.**001**	6.93	1.82	26.36	**0**.**005**

### The result of univariate and multivariate analysis for prolonged hospital stay patients

We used univariate logistic analysis to identify variables associated with a prolonged hospital stay in patients who underwent TKA, and 19 predictive factors were analyzed. The results showed that 14 predictive factors were associated with a prolonged hospital stay in patients who underwent TKA. Our study showed that Age, Hb, ASA Status, Operation Duration, Race, Type of Anaesthesia, Procedure Description, D.M., CHF, CVA, Creatinine, Day operation, Blood transfusion, and reoperation were the predictor risk factors for prolonged hospital stay in patients who underwent TKA (Table 2).

The multivariate logistics regression model was also performed to screen for independent risk factors. The results showed that Age (*P* ≤ 0.001), Hb (*P* ≤ 0.001), Operation Duration in mins (*P* ≤ 0.001), Procedure Description (*P* ≤ 0.001), D.M. (*P* ≤ 0.001), Day operation (*P* ≤ 0.001), Blood transfusion (*P* ≤ 0.001), Repeat Op within 30 days (*P* ≤ 0.001) were independent prognostic factors of prolonged hospital stay (Table 2).

### Development of the prediction model and evaluation of the accuracy of the nomogram in the training group

**Figure 2:**
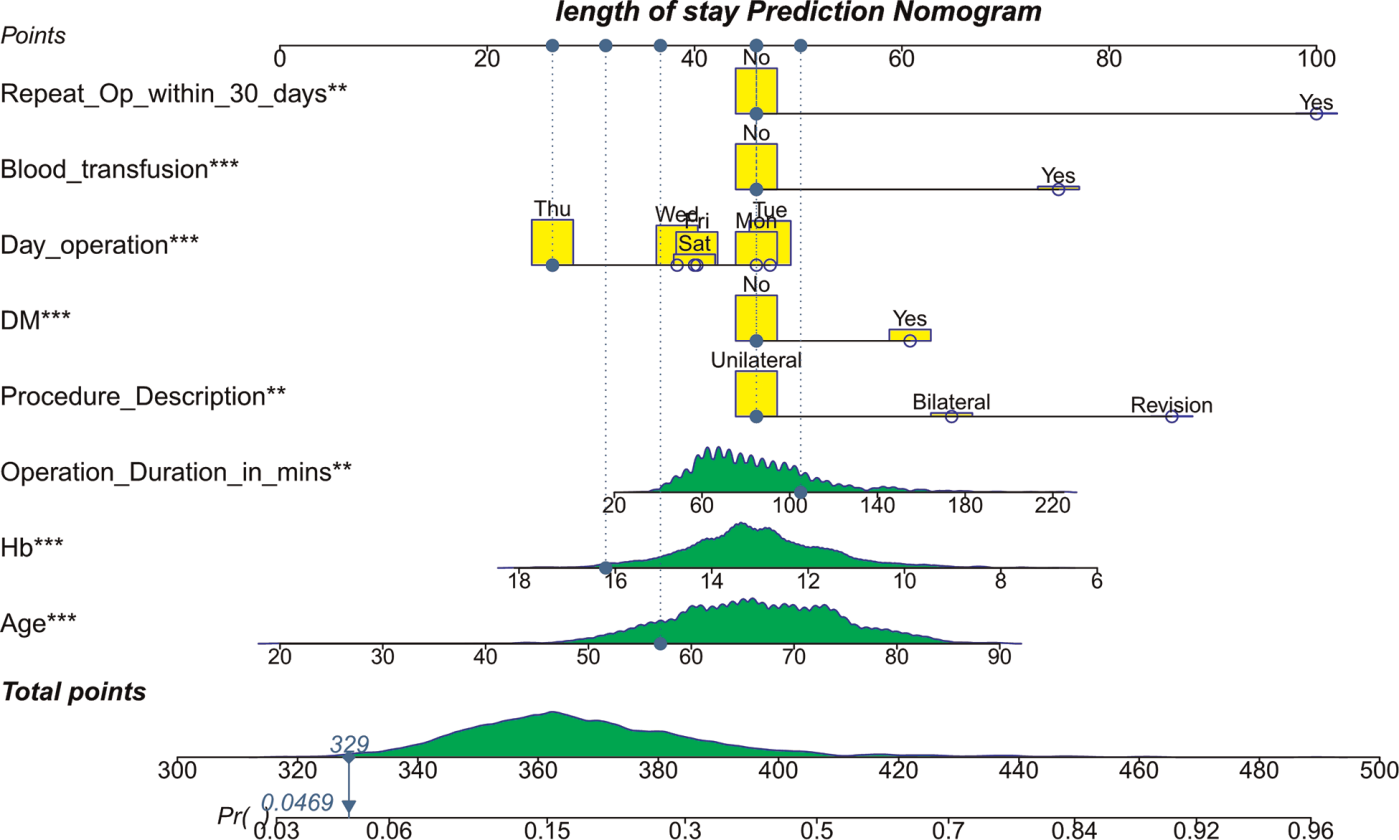
The nomogram for a prolonged hospital stay in undergoing TKA patients.

**Figure 3:**
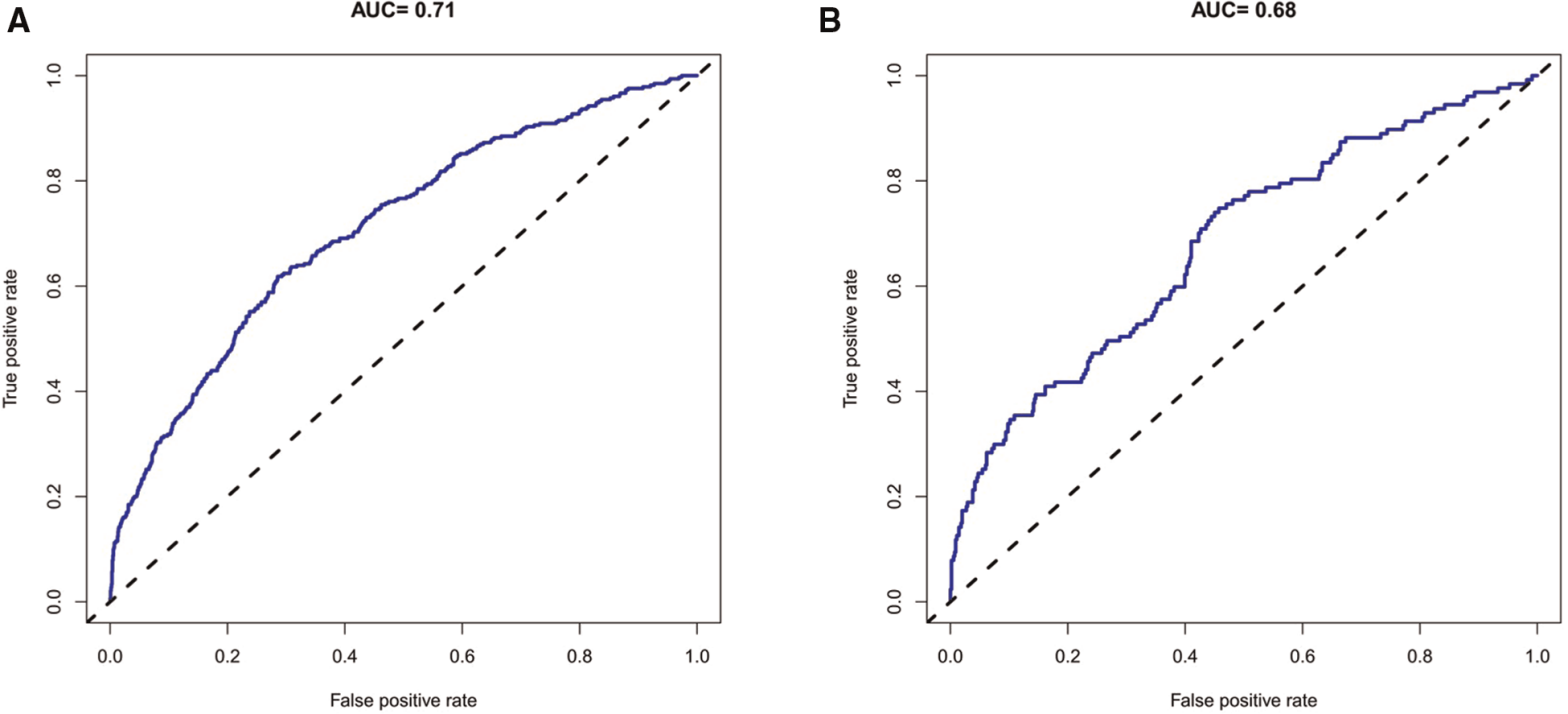
The ROC curves of prolonged hospital stay in the training set (**A**) and validation set (**B**).

**Figure 4:**
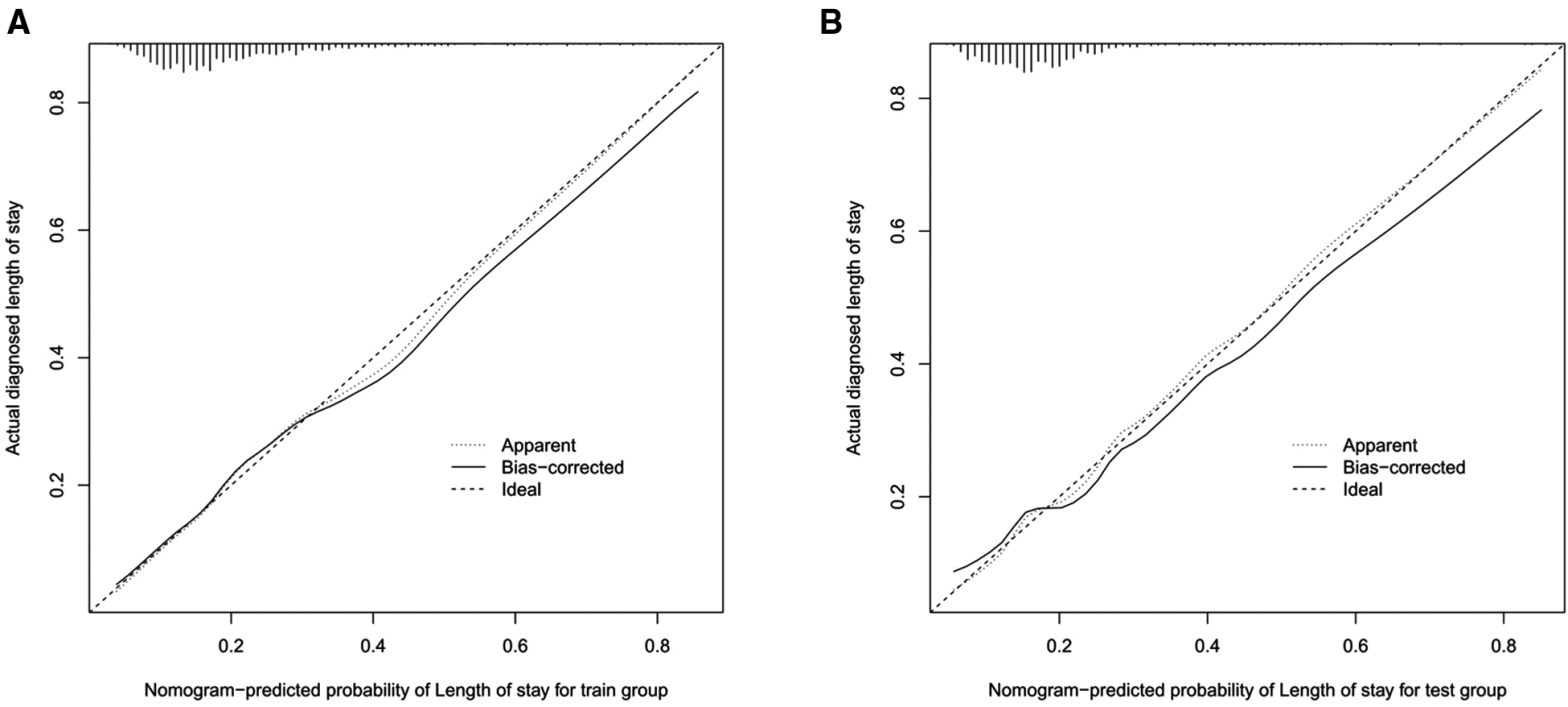
The Calibration curves of prolonged hospital stay in the training set (**A**) and validation set (**B**).

**Figure 5:**
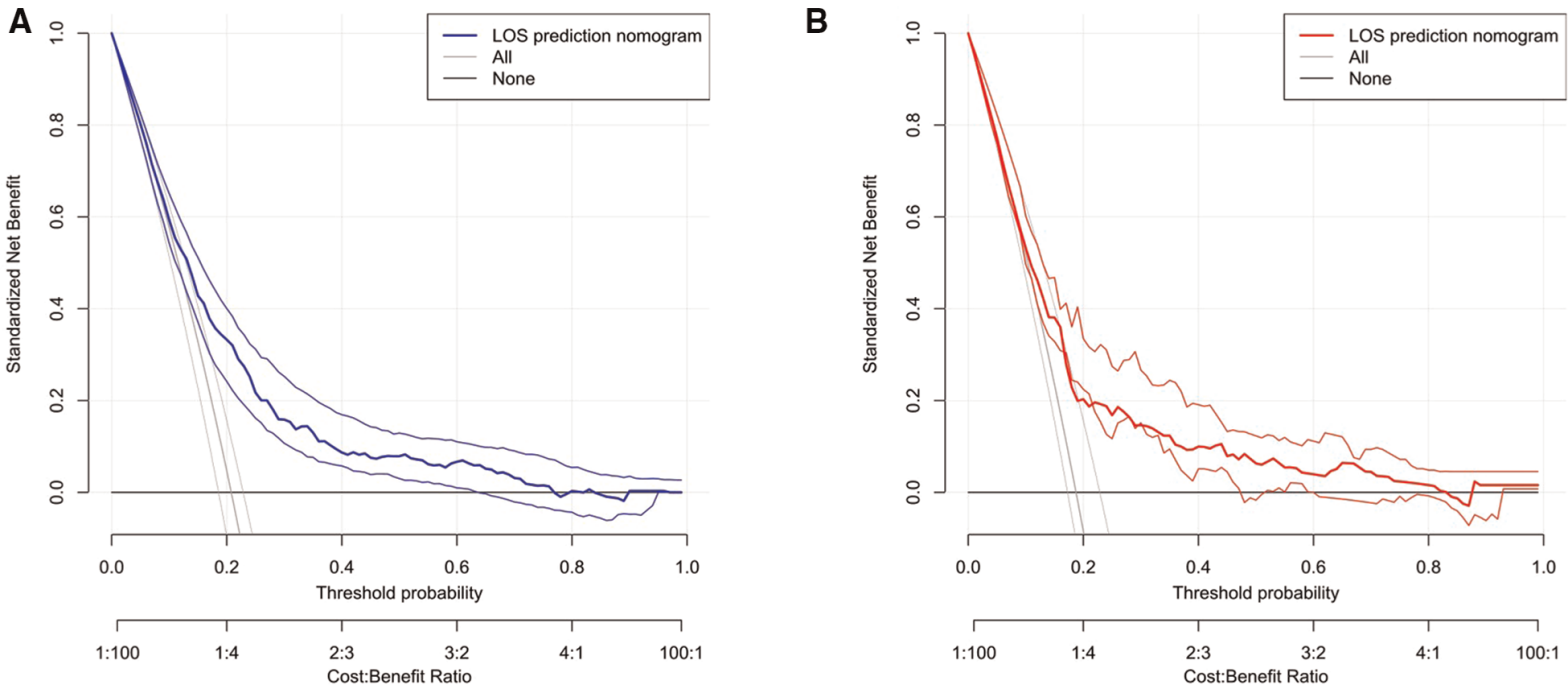
The DCA curve of prolonged hospital stay in the training set (**A**) and validation set (**B**).

Based on eight independent risk factors, we established a nomogram for prolonged hospital stay in TKA patients (Figure 2). Meanwhile, the area under the ROC curve was 0.710 in the training group (Figure 3A). This indicated that the prediction model established based on the results of multivariate logistics regression analysis had a high predictive value. In addition, we also plotted the calibration curve and DCA for the nomogram in the training sets. The results showed that the nomogram could be a good tool for predicting prolonged hospital stay in TKA patients (Figures 4A, 5A).

### Evaluating the accuracy of the nomogram in the validation group

The ROC curve shows that the rosette has high predictive performance, and its AUC is 0.680 (Figure 3B). It indicates that the prediction model has a strong prediction ability. The calibration curves used to predict the prolonged hospital stay in the validation cohort showed good consistency and prediction ability (Figure 4B). The DCA of prolonged hospital stay indicated that this nomogram could be an excellent diagnostic tool for patients undergoing TKA (Figure 5B).

## Discussion

Our study aimed to develop and validate a prolonged hospital stay risk prediction model for TKA patients. The risk factors were Age, Hb, Operation Duration, Procedure Description, D.M., Day operation, Blood transfusion, and Repeat Operation within 30 days. We built the nomogram using the above risk factors for prolonged hospital stay in TKA patients. The nomogram transforms the complex regression equation into a visual graph, making the prediction model's results more readable and convenient for clinicians. Because of the intuitive and easy-to-understand characteristics of the nomogram, it has gradually received more attention and has been applied in medical research and clinical practice.

Our study found that age was an independent risk factor for prolonged hospitalization after TKA. This might be because elderly patients are at high risk and prone to many diseases. Factors such as poor autoimmune ability, health status, and other reasons, coupled with the increased risk of infection due to blood loss during surgery, contribute to increasing their hospital stay. Davide Tornese et al. (15) found that the average LOS was 5.08 ± 2.52 days in the Department of Orthopedic Surgery, and the age was predictive of a more extended stay. In Corentin Roger et al. (16) study, the predictors of LOS were identified using a survival model that considered age as a continuous variable, individual comorbidities, and the discharge destination.

This study shows that preoperative hemoglobin is related to hospital stay. The hospitalization time decreased with the increase in hemoglobin value (17). We suggest that anemia be corrected before the operation to shorten hospital stays. Raut et al. (18) found a negative correlation between the level of Hb and LOS before and on the first day after the operation. However, there was still no correlation between the level of Hb decline and LOS, emphasizing the importance of improving the level of Hb before the operation.

Similarly, the longer the operative time, the higher the risk of prolonged patient hospitalization. Cregar (19) and Garbarino (20) indicated that less time spent in the operating theatre could lead to shorter LOS for revision and primary TKA patients. Lu (21) found that Total knee arthroplasty (TKA) frequently results in significant blood loss with accompanying hemoglobin loss and potentially increased transfusion rates. Unfortunately, transfusions have associated risks, including postoperative confusion, infection, cardiac arrhythmias, fluid overload, prolonged hospital stay, and increased mortality.

The highest risk score in the independent predictor was blood transfusion, many studies have come to the same conclusion, and the transfusion rate was directly related to the hospital stay (22–24). Autologous blood transfusion is often required when the blood loss is between 1000 and 1500 ml. Research shows that the total blood loss after TKA may be as high as 2000 ml, and the proportion of blood transfusion may be as high as 67% (25, 26). In a cohort of 228,316 TKA patients at 922 hospitals, the mean predicted probability of TKA transfusion was 7.9%, with 60% (95% CI, 36%–87%) of patients having hospital stays of more than three days (22). In a cross-sectional study of 4,544,999 patients who received TKA between January 2000 and December 2009, blood transfusions were associated with in-hospital mortality, and hospital stay increased by 0.71 ± 0.01 days (23). In addition, Danninger et al. (24) showed a significantly higher rate of significant complications in patients receiving transfusion (19.1% vs. 11.2%, *P* < 0.0001), and the mean length of hospital stay was significantly increased.

Our study found that the operation date was a significant factor in predicting the risk of hospital stays after blood transfusion. Similarly, studies demonstrated that operation procedures or factors related to doctors and nurses provide clinically relevant improvements in explaining hospital stay and patient-related risk factors (7). In a prospective cohort study of 4,509 patients who underwent initial TKA at four hospitals between January 1, 2016, and September 30, 2017, who received surgery later in the day were predicted to have a more extended hospital stay, with patients who had surgery on Friday having a significantly longer hospital stay than patients who had surgery on Monday (7). Our study showed that the risk prediction scores of operations on Monday, Tuesday, and Friday were much higher than those on Thursday. The length of hospital stay was related to operation data. Possible reasons for this result include patients coming from different countries, surgical hospital systems, and doctors' moods and preferences.

Diabetes is a chronic disease that can lead to multiple systemic comorbidities. This study found that the higher the comorbidity index, the longer the hospital stays were. Swain et al. (27) showed that 67% of patients with osteoarthritis had at least one other chronic condition, 20% more than those without osteoarthritis. The DM was the independent predictor of prolonged hospital stay. Similarly, other previous studies have suggested that TKA patients with comorbidities had prolonged hospital stays (28–31). Higuera et al. (30) showed that chronic heart failure was associated with extended hospital stays and increased rates of major postoperative complications in TKA patients. In a study of 15,321 TKA patients, 18.2% had a medical comorbidity D.M., with a 300% increase in overall mortality. Belmont et al. (31) found that DM was an independent predictor of hospitalization for four days or more.

Our study found that unilateral TKA under epidural anesthesia is economical, efficient, and has an ideal surgical effect for patients with osteoarthritis. For patients with bilateral knee arthritis, a comprehensive health assessment should be conducted before surgery. For high-risk patients, especially those with severe cardiovascular diseases, simultaneous bilateral total knee replacement should be avoided as far as possible (32–35). The reasons for reoperation TKA were that the patients had cardiac complications, pulmonary complications, and renal and cerebrovascular complications before the operation. The local complications of patients after operation include non-infection-related complications, wound infection, and peripheral nerve injury. Furthermore, we also found that the causes of revision surgery were periprosthetic infection, aseptic loosening, osteolysis, abrasion, joint dislocation, periprosthetic fracture, and patellar-related complications. These factors will lead to prolonged hospitalization (16, 36–38).

There are still some limitations to our study. First, this study is retrospective, using clinical data from a single center. Therefore, there may be differences in treatment strategies and race, etc. Second, because of differences in healthcare settings and practices, the predictive models developed in one country are unlikely to be directly applicable in another, requiring external validation and an updating of the predictive performance of models in other new patients. Finally, some influencing factors may not be included in this paper, including patient income, medical expenses, medical insurance, hospital location, etc. Potential factors not included may also have some influence on the results.

## Conclusion

To make better use of these factors and identify patients' risk factors early, the medical team should plan a patient's rehabilitation path as a whole. Advantages of this approach include better resource allocation, maximizing medical resources, improving the functional recovery of patients, and reducing the overall cost of hospital stay and surgery. We also hope that these results will help clinicians in the future.

## Data Availability

The datasets presented in this study can be found in online repositories. The names of the repository/repositories and accession number(s) can be found below: https://datadryad.org/stash/dataset/doi:10.5061/dryad.73250.
